# Interaction between bacteria and cholesterol crystals: Implications for endocarditis and atherosclerosis

**DOI:** 10.1371/journal.pone.0263847

**Published:** 2022-02-18

**Authors:** Manel Boumegouas, Manjunath Raju, Joseph Gardiner, Neal Hammer, Yehia Saleh, Abdullah Al-Abcha, Apoorv Kalra, George S. Abela

**Affiliations:** 1 Department of Medicine, Division of Cardiology, Michigan State University, East Lansing, Michigan, United States of America; 2 Department of Epidemiology and Biostatistics, Michigan State University, East Lansing, Michigan, United States of America; 3 Department of Microbiology and Molecular Genetics, Michigan State University, East Lansing, Michigan, United States of America; 4 Department of Cardiology, Houston Methodist DeBakey Heart & Vascular Center, Houston, Texas, United States of America; 5 Department of Medicine, Division of Internal Medicine, Michigan State University/Sparrow Hospital, Lansing, Michigan, United States of America; 6 Metro Infectious Disease Consultants, Kansas City, Missouri, United States of America; 7 Division of Pathology, Department of Physiology, Michigan State University, East Lansing, Michigan, United States of America; Thomas Jefferson University, UNITED STATES

## Abstract

**Background:**

The interaction between pathogenic bacteria and cholesterol crystals (CCs) has not been investigated. However, CCs are found extensively in atherosclerotic plaques and sclerotic cardiac valves. Interactions between pathogenic bacteria and CCs could provide insights into destabilization of atherosclerotic plaques and bacterial adhesion to cardiac valves.

**Methods:**

*Staphylococcus aureus* and *Pseudomonas aeruginosa* were used to assess *in vitro* bacterial adhesion to CCs and proliferation in the presence of CCs compared to plastic microspheres and glass shards as controls. *Ex vivo* studies evaluated bacterial adhesion to atherosclerotic rabbit arteries compared to normal arteries and human atherosclerotic carotid plaques compared to normal carotid arteries. Scanning electron microscopy (SEM) was used to visualize bacterial adhesion to CCs and confocal microscopy was used to detect cholesterol binding to bacteria grown in the presence or absence of CCs.

**Results:**

*In vitro*, *S*. *aureus* and *P*. *aeruginosa* displayed significantly greater adhesion, 36% (p<0.0001) and 89% (p<0.0001), respectively, and growth upon exposure to CCs compared to microspheres or glass shards. Rabbit and human atherosclerotic arteries contained significantly greater bacterial burdens compared to controls (4× (p<0.0004); 3× (p<0.019), respectively. SEM demonstrated that bacteria adhered and appeared to degrade CCs. Consistent with this, confocal microscopy indicated increased cholesterol bound to the bacterial cells.

**Conclusions:**

This study is the first to demonstrate an interaction between bacteria and CCs showing that bacteria dissolve and bind to CCs. This interaction helps to elucidate adhesion of bacteria to sclerotic valves and atherosclerotic plaques that may contribute to endocarditis and plaque destabilization.

## Introduction

Bacteria residing in atherosclerotic plaques has been reported but their role in atherosclerosis has been unclear. Studies have focused primarily on a causal relationship between bacteria and atherosclerotic plaque [1]. Despite evidence implying a bacterial-plaque interaction, inflammation triggered by cholesterol crystals (CCs) in the absence of detectable bacteria also promotes atherosclerosis further complicating a clear connection between bacterial colonization and arterial blockage [2, 3]. Moreover, treatment with antibiotics in patients with acute cardiovascular disease did not provide a benefit [4]. However, systemic infections have been associated with subsequent myocardial infarction [5, 6]. We previously discovered that as cholesterol undergoes a phase change from a liquid to a crystalline state it occupies a greater volume. This can cause volume expansion within the lipid core leading to perforation of the fibrous cap and destabilization of the atherosclerotic plaque [7, 8]. A similar finding of CCs perforating the valve surface was observed in human sclerotic valves and in atherosclerotic rabbit valves [9, 10]. In human coronary arteries during myocardial infarction scavenger cells, primarily macrophages were present engulfing, degrading, and binding to CCs [11]. Since bacteria also scavenge host-derived metabolites [12, 13], we conducted this study to determine whether an interaction between bacteria and CCs is observed. To test this, we evaluated Gram positive and negative bacterial growth as well as adhesion to CCs in *in vitro* and *ex vivo* models.

## Materials and methods

### *In vitro* studies

Initial studies were conducted *in vitro* to evaluate the growth and adhesion characteristics of clinical strains of *Staphylococcus aureus* (ATTC #43300) and *Pseudomonas aeruginosa* (ATTC #27857) to CCs. Crystals were synthesized by dissolving cholesterol powder in methanol followed by evaporation as previously described [14]. To validate *S*. *aureus* and *P*. *aeruginosa* adherence to CC was specific and not related to the shape or hardness, we used plastic microspheres (250 μm) and ground glass shards as controls glued to coverslips. For adhesion studies all particulates (CC, glass shards, plastic microspheres, 2.5mg) were attached to glass coverslips covered with epoxy resin which was also tested independently.

Overnight cultures of *S*. *aureus* and *P*. *aeruginosa* were diluted 10-fold in Mueller Hinton Broth (MHB). The suspensions were further incubated for 2 hours on an oscillating shaker. Bacterial concentration at this time (T1) was quantified using the spread plated method below as starting concentration. Next, glass coverslips containing the indicated particulates were incubated at 37°C in a Petri dish containing 2 ml of bacterial suspension at T1 and 20 ml of buffered saline. The Petri dishes were incubated for 1, 2, 3, and 4 h on an oscillating shaker. Coverslips were then removed from the Petri dish washed 3 times and crushed in MHB creating a suspension. Quantification of bacterial cells adhered to the particulates was performed using spread plate methods as below.

For growth studies, overnight suspensions of *S*. *aureus* or *P*. *aeruginosa* were adjusted to 0.5 Mcfarland concentration. Five ml of 1:20 dilution of 0.5 Mcfarland suspension in MHB was prepared. Six replicates with (2.5 mg of CCs) and without CCs were incubated at 37°C in oscillating shaker for 3, 4, 5 and 6 h. Bacterial quantification was performed using spread-plate method as below.

Spread plate method: 10 μl of serial 10-fold dilutions of MHB suspension was spread on agar plate using a sterilized spreader. Agar plate was incubated at 37°C overnight and bacterial colonies were counted manually the next morning. This raw count was multiplied by dilution factor and adjusted for weight of CCs and represented as CFU/mg of CCs.

### *Ex vivo* rabbit arteries

Another set of experiments were conducted *ex vivo* to evaluate whether arteries containing CC enriched atherosclerotic plaques enhance bacterial proliferation using arteries from an atherosclerotic rabbit model compared to normal arteries from non-atherosclerotic rabbits. Ten rabbits were made atherosclerotic by balloon de-endothelialization via femoral cutdown under general anesthesia using ketamine (50 mg/kg, i.m.) and xylazine (10 mg/kg, i.m.) once for a single procedure followed by feeding a cholesterol enriched diet alternating with normal chow for six months [15]. Buprenorphine (0.01mg/kg) was injected subcutaneously every 12 hours for 24 hours following the femoral cutdown to alleviate any discomfort. After euthanasia using a combination of pentobarbital (390 mg/ml) and phenytoin (50 mg/ml) administered at a dose of 100 mg/kg of pentobarbital via the marginal auricular vein, arterial tissue samples were then obtained from the aortas and placed in a washer ring that only exposed the intimal surface of the artery to a broth solution with *S*. *aureus*. The same was repeated for normal control rabbits fed normal rabbit chow without intimal injury. Ten samples were obtained from each of 5 atherosclerotic rabbits and 5 normal controls. For each group, five samples were incubated for 1 h and another five samples were incubated for 3 hours. Arterial segments (normal and atherosclerotic) were then removed, washed with PBS and pulverized in an Eppendorf tube with micro-pestle and resuspended and 10 μl of serial 10-fold dilutions of suspension was spread on agar plate using sterilized spreader. These were then incubated overnight at 37°C and bacterial colonies counted the following morning. Bacterial colony counts were measured at each time interval and SEM used to examine for presence of bacteria on the arterial samples.

Rabbits were housed in specifically designated unit and supervised by a veterinarian. They were monitored daily and provided adequate feed and water and cages cleaned on a regular basis by technical staff. Nesting materials and hay were provided for enrichment and engagement. This protocol was approved by Michigan State University’s Animal Care and Use Committee following National Institute of Health guidelines (Institutional Animal Care and Use Committee # 03/18-034-01).

### *Ex vivo* human arterial plaque

Another set of experiments were conducted *ex vivo* to investigated bacterial growth on human carotid arterial plaques rich in CCs compared to non-atherosclerotic carotid arteries. Seven de-identified atherosclerotic carotid plaques were obtained from the operating room during carotid endarterectomy. These were matched with seven normal carotid arteries obtained from autopsy within 48 hours of death. Arterial tissue samples were placed in a washer ring exposing only the intima as described above and then placed in broth solution with *S*. *aureus*. The same was repeated for the normal control arteries. For each group, seven samples were incubated in broth for 1 h and then washed and processed for bacterial colony counts as above. Bacterial presence on the intimal surface was examined by SEM. Both Michigan State University and Sparrow Hospital institutional review boards approved this protocol (# 0518-exempt and no consent was required).

#### Scanning electron microscopy

For *in vitro* studies, samples were aliquoted from runs of CCs with and without bacteria, fixed in 10% buffered formalin and examined by scanning electron microscopy (SEM). Aliquots were then placed on stubs and air dried for 48 hours. For *ex vivo* studies, 5 mm segments of arterial tissue samples were fixed in 10% buffered formalin, air dried and placed on stubs [7, 8]. The stubs are then coated with a NEOC-AT osmium plasma coater (Meiwafosis Co, Ltd., Osaka, Japan) and examined using a JEOL SEM (JEOL 6610LV SEM, JEOL Ltd., Tokyo, Japan).

#### Light microscopy

Carotid arterial plaque samples were fixed in 10% buffered formalin, embedded in paraffin blocks, serially sectioned and mounted on glass slides. These were then stained with trichrome and examined and photographed with a light microscope.

#### Confocal microscopy

*S*. *aureus* bacteria were incubated with and without CCs for 3 h at 37°C in broth. After incubation, samples were fixed with 4% glutaraldehyde and then stained for cholesterol using a green, fluorescent dye (cholesteryl BODIPY-C12, Invitrogen, Eugene, OR) at a 1/100 dilution (75% ethanol) in a test tube for 3 minutes [7]. Samples were then transferred to a slide incubator chamber and visualized. Confocal fluorescence images of the bacteria were acquired using a Zeiss Pascal LSM microscope (Carl Zeiss, Inc, Jena, Germany). The green fluorescence was excited with the 488 nm argon laser line, and emission was collected using a 505 to 530 nm band-pass filter.

#### Statistical analysis

Because the distributions of *S*. *aureus* and *P*. *aeruginosa* adhesion data were skewed, a Box-Cox transformation was applied to mitigate skewness. We used two-way analysis of variance (ANOVA) to the transformed data with factors GROUP (levels: plastic microspheres, CCs, glass shards, glue) and HOUR (levels: 1, 2, 3, 4). Six replicate observations were available for each of the 16 independent experiments. The crossed effect (GROUP×HOUR) was not significant. We estimated group-specific least-squares means, 95% confidence intervals and assessed the significance of differences between estimates.

Two parallel groups (CC and Control) were used in the *S*. *Aureus* and *P*. *aeruginosa* growth studies with outcomes at 3, 4, 5 and 6 hours, and six replicate observations. A relative response was defined as the ratio of the outcome to the fixed baseline (hour 0) value. A log transformation was performed and the relative responses at hours 3, 4, 5 and 6 were analyzed as correlated data with group and hour as factors in a repeated measures analysis.

The rabbit artery study was designed with two groups (normal and atherosclerotic), five replicates per group and two incubation periods (hour 1, hour 3). A Box-Cox transformation was applied to the 20 bacterial colony counts. Responses at hour 1 and 3 were analyzed as correlated data with group and period as factors.

In the human arterial studies bacterial counts were Box-Cox transformed and compared between atherosclerotic and normal carotid samples by t-test.

Analyses were conducted in SAS software, ver 9.4 (SAS Institute Inc, Cary, NC).

## Results

### *In vitro* bacterial adhesion studies

**Fig 1:**
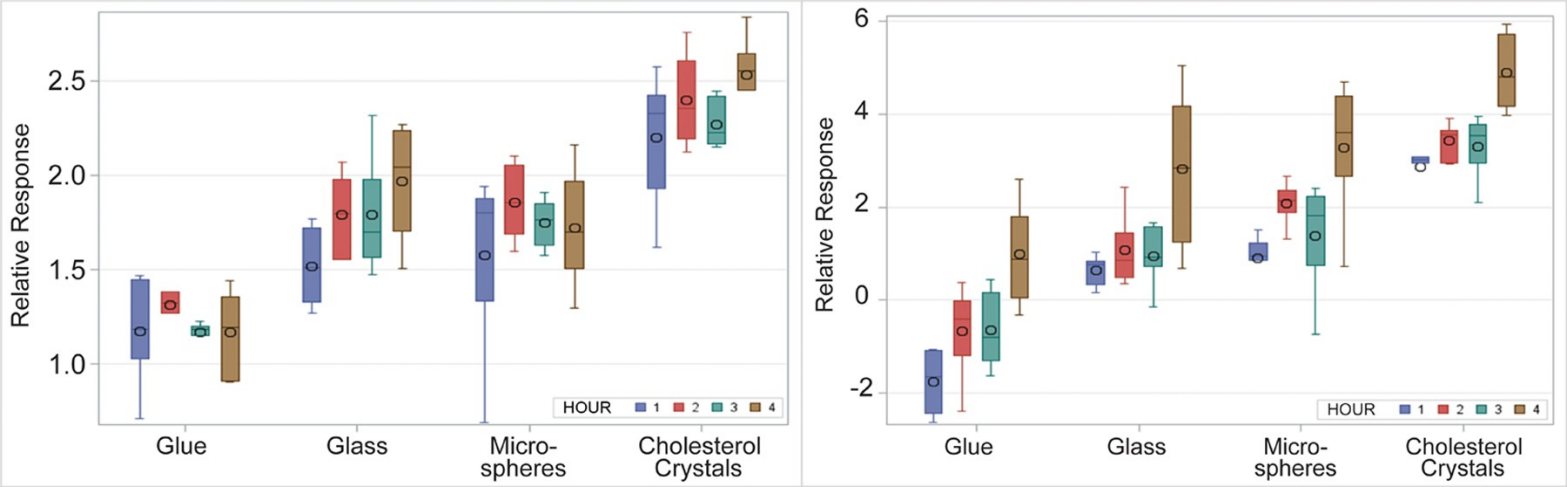
*In vitro* bacterial adhesion. (left) Box-Cox transformed graph of *S*. *aureus* bacterial count with glue, glass, plastic microspheres, and cholesterol crystals (p<0.0001). (right) Box-Cox transformed graph of *P*. *aeruginosa* bacterial count demonstrating highest count with cholesterol crystals (p<0.0001). P-values were obtained from analysis of variance and adjusted for multiplicity by the Bonferroni method.

Bacterial colony counts of *S*. *aureus* and *P*. *aeruginosa* were highest upon incubation with CCs compared to glass shards, microspheres, and glue (S1 and S2 Tables in S1 File). After Box-Cox transformation of bacterial count, significant differences were found (p<0.0001) between groups except for the glass and microsphere pair (Fig 1). Bacterial adhesion was also significantly greater for CCs for each time interval compared to controls for both *S*. *aureus* and *P*. *aeruginosa*. CCs vs. microspheres: 0.63, 95% CI:0.48,0.78 for SA; p<0.0001 and 1.71,95% CI:1.19, 2.22 for PA; p<0.0001). S3 Table in S1 File summarizes the statistical comparison based on the transformed data.

### *In vitro* bacterial growth studies

**Fig 2:**
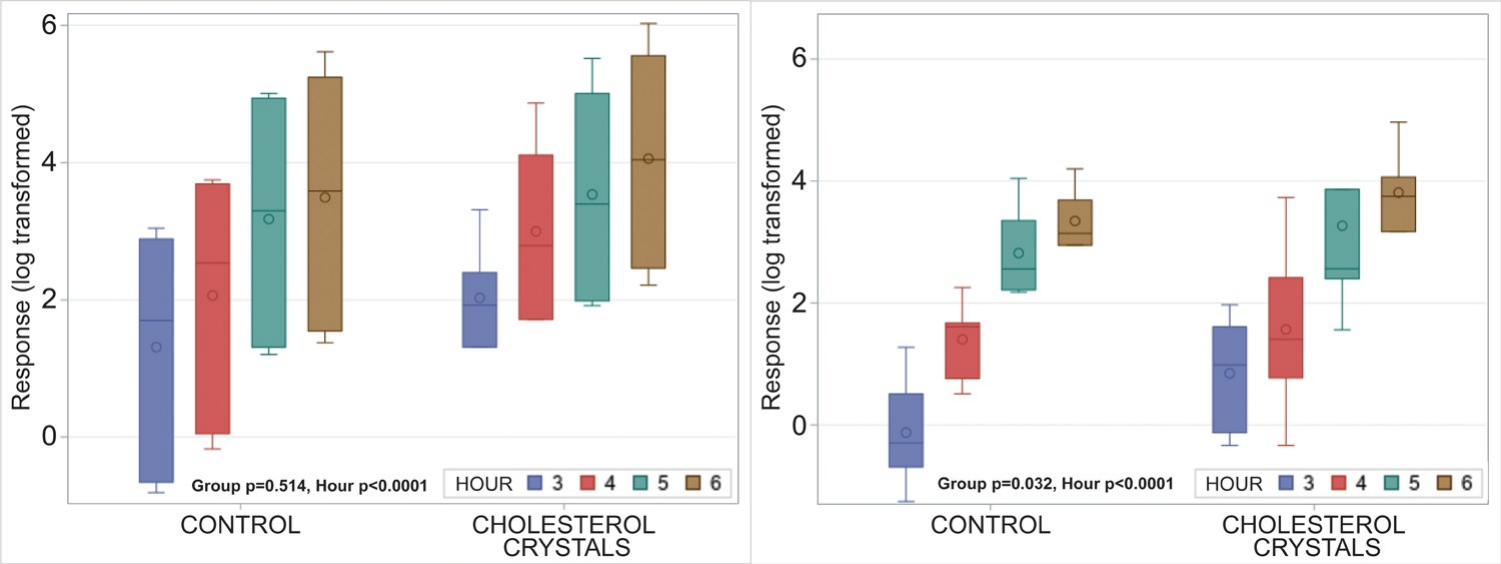
*In vitro* bacterial growth. Log-transformed relative response of bacterial colony count with cholesterol crystals and control at 3, 4, 5 and 6 hours of incubation. Left panel, *S*. *aureus*; Right panel *P*. *aeruginosa*. P-values were obtained from t-tests in repeated measures analyses. For both studies the time effect was significant (P<0.0001). Group effect: P = 0.514 for *S*. *aureus*, and P = 0.032 for *P*. *aeruginosa*.

For *S*. *aureus*, CCs compared to control was elevated but the ratio was not significant, 1.90 (95% CI: 0.23, 15.91), p = 0.514. For *P*. *aeruginosa*, CCs compared to control was elevated and significant, 1.67 (95% CI: 1.05, 2.64), p = 0.032. However, growth over time in both studies was significant (3 DF, p<0.0001). (Fig 2 and S4 Table in S1 File).

### *Ex vivo* rabbit arteries

**Fig 3:**
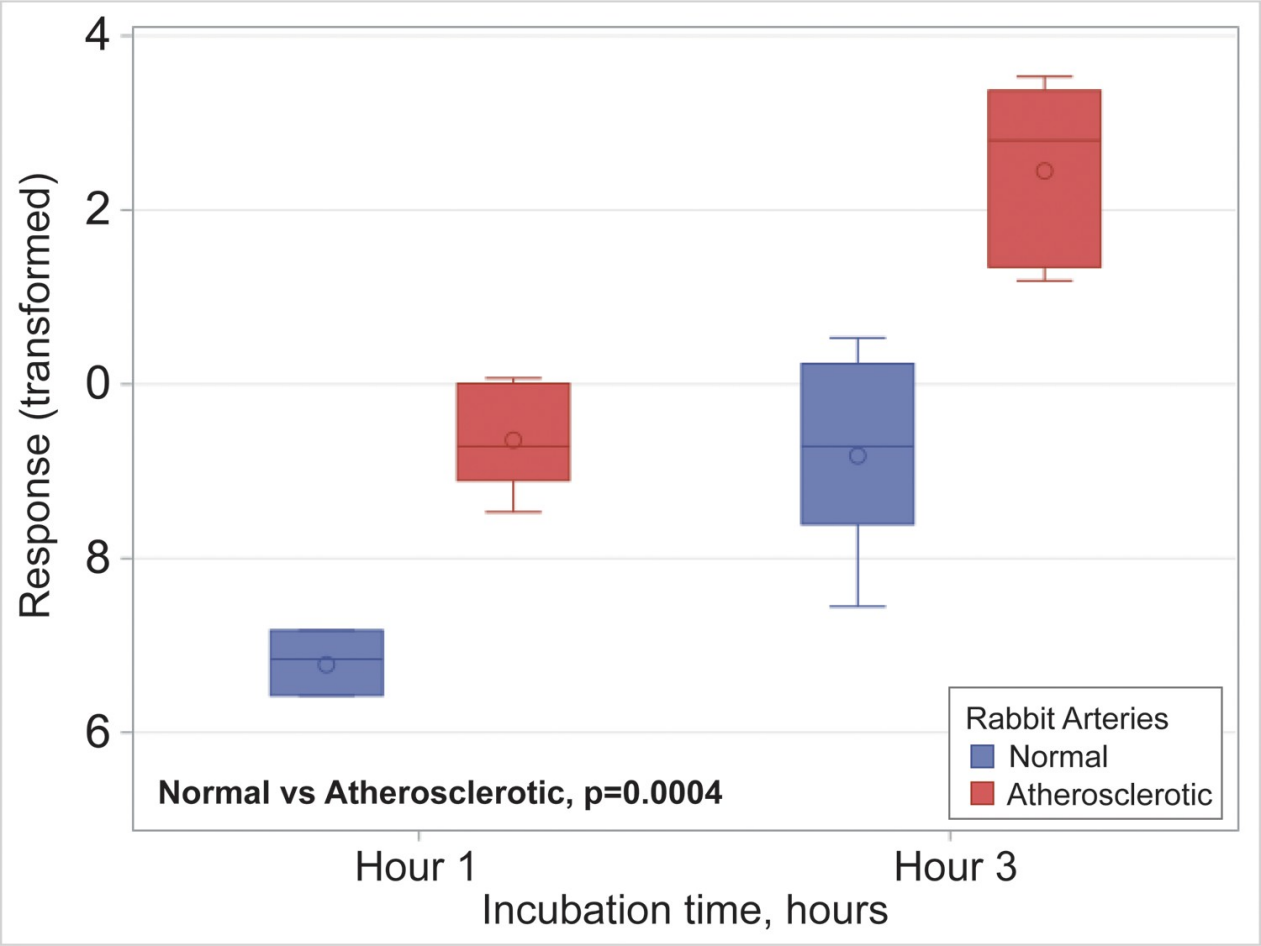
*Ex vivo* bacterial growth in rabbit arteries. Bacterial growth graph with bacterial counts in normal control and atherosclerotic rabbit arteries using Box-Cox transformation. P- value was obtained from t-test in a mixed effects model with group and hour as factors.

Rabbit arteries demonstrated that atherosclerotic samples had a 4 fold increased bacterial counts compared with the normal control arteries (S5 Table in S1 File). After Box-Cox transformation of bacterial count there was a significantly increased number of bacteria present in atherosclerotic arteries compared with normal controls at one hour incubation (2100 ± 815.5 vs. 500 ± 418.3, p<0.0004) (Fig 3).

### *Ex vivo* human arterial plaque

**Fig 4:**
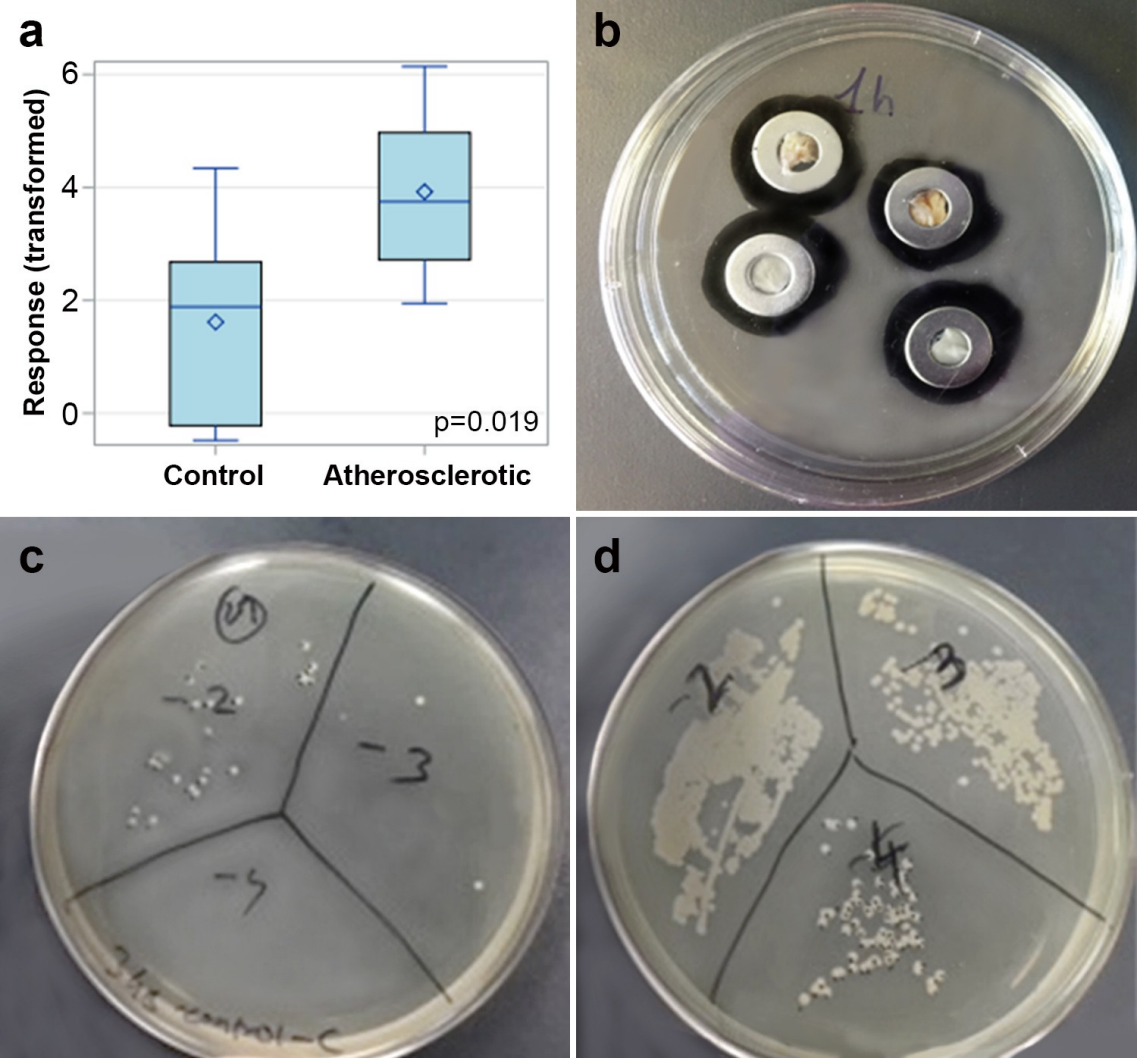
*Ex vivo* bacterial growth in human arterial plaques. (a) Graphic of bacterial counts of normal control and atherosclerotic human arterial plaques using Box-Cox transformation. P-value was obtained from a two-sample t-test. (b) The arteries embedded in a washer ring exposing the intimal surface and covering the back of the washer. (c) Bacterial count in human normal carotid tissue comparted to (d) bacterial count in atherosclerotic carotid tissue.

The mean bacterial count was 3 times higher in atherosclerotic arterial plaques compared to ‘normal’ controls (S6 Table in S1 File). After Box-Cox transformation of the bacterial count a significant difference was observed in atherosclerotic arterial plaques compared to normal controls (18.4 ± 13.0 vs. 5.9 ± 6.4, p<0.019), (Fig 4).

#### Microscopy

**Fig 5:**
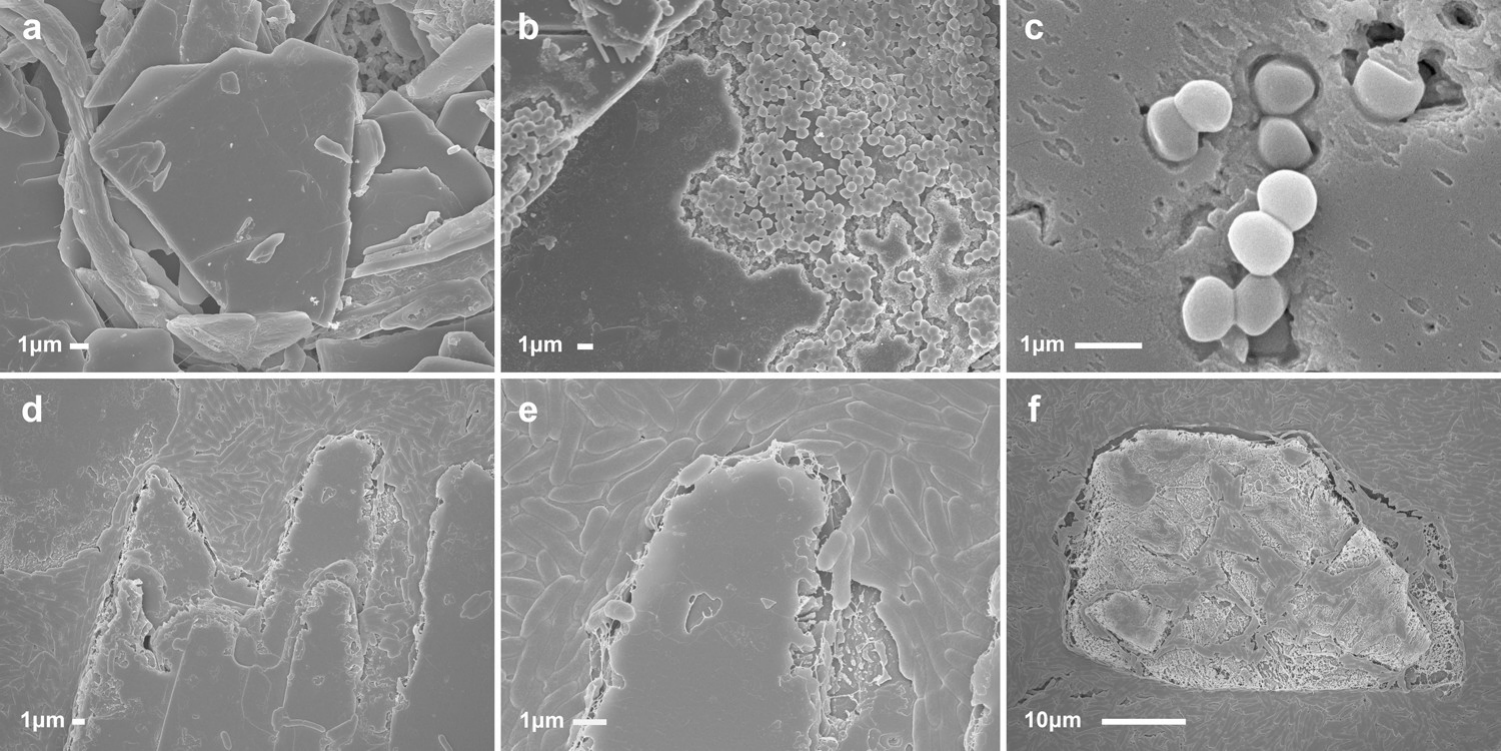
Scanning electron micrograph of bacteria engaging cholesterol crystals. *In vitro S*. *aureus incubated with cholesterol crystals (top)*: (a) Cholesterol crystals incubated in broth without bacteria as control (b) Staphylococcus bacteria engulfing and degrading crystal after 2.5 hours incubation. (c) Staphylococcus bacteria noted engaging and punctating the crystal surface at 1 hour incubation. *In vitro P*. *aeruginosa incubated with cholesterol crystals (bottom)*: (d) *Pseudomonas* bacteria seen engulfing and eroding crystals forming wedges into the crystal body (e) higher magnification demonstrates the detail of the bacterial and the crystal erosion with loss of crystal sharp edges; (f) another example of eroding crystal with bacteria above and around the crystal.

**Fig 6:**
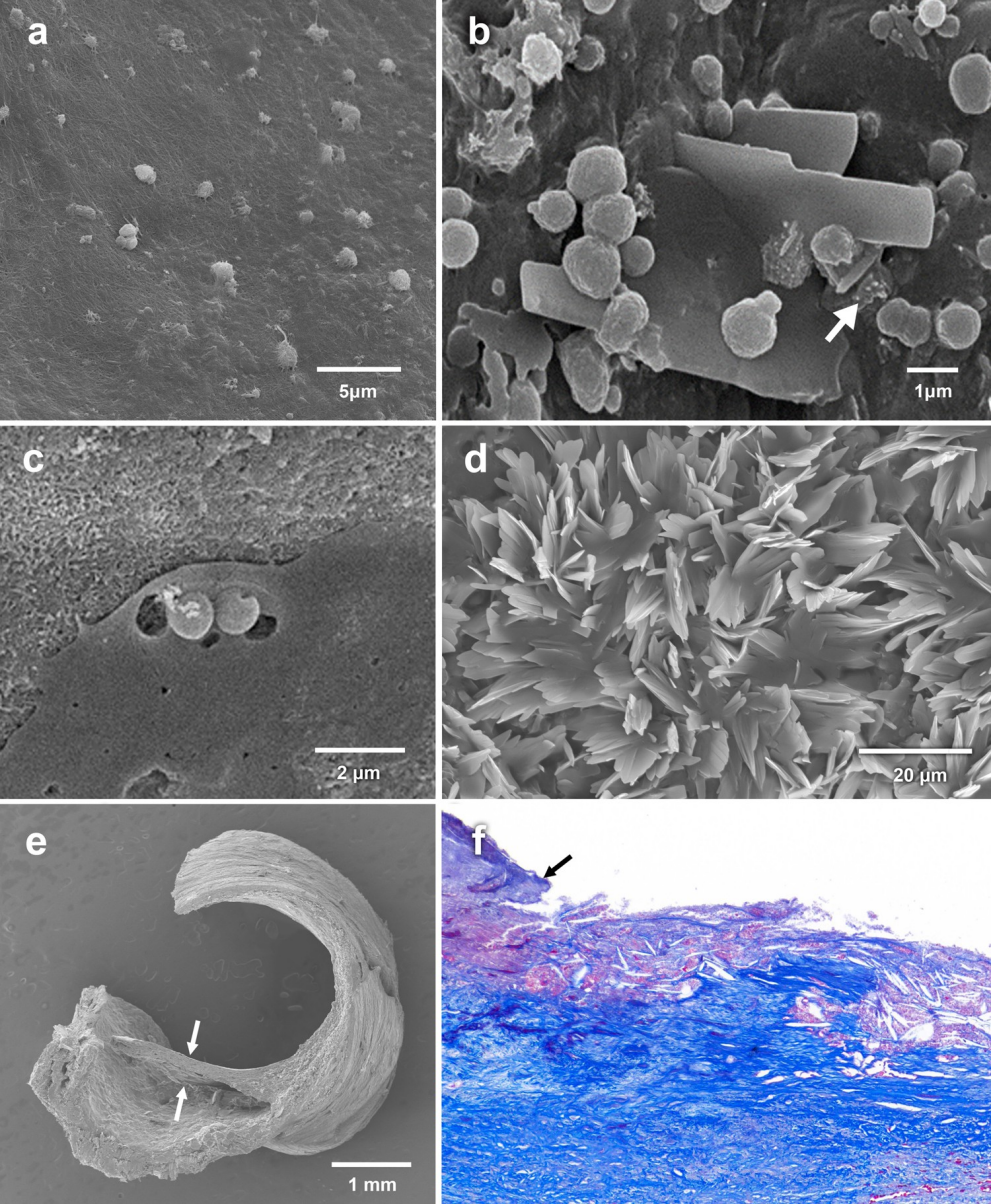
Micrographs of *ex vivo S*. *aureus* bacteria on human plaques. (a) Scanning electron micrograph of normal carotid artery with few bacteria attached to intimal surface; (b) atherosclerotic carotid plaque with many bacteria and macrophages (white arrow) attached to cholesterol crystals; (c) bacteria attached and degrading crystal in human plaque; (d) Scanning electron micrograph demonstrating extensive sheets of cholesterol crystals covering the plaque surface with absence of any fibrous cap at this advanced stage of plaque disruption; (e) Example of an intact fibrous cap (arrows) from a human carotid plaque; (f) Light microscopic image of a completely ruptured and eroded plaque with cholesterol crystals filling the base and a remnant insertion site of a fibrous cap (arrow).

*Scanning electron microscopy*. By SEM both *S*. *aureus* and *P*. *aeruginosa* were found to be degrading CCs. Additionally, *S*. *aureus* were found attached to CCs (Fig 5), and on CCs in atherosclerotic rabbit and human carotid arteries specimens (Fig 6). SEM of the human carotid plaques reveals sheets of CCs with absence of fibrous caps.

*Light microscopy*. The carotid artery plaques were eroded without fibrous caps exposing their lipid core and crystals (Fig 6). This is the surface that was exposed to the bacterial broth.

**Fig 7:**
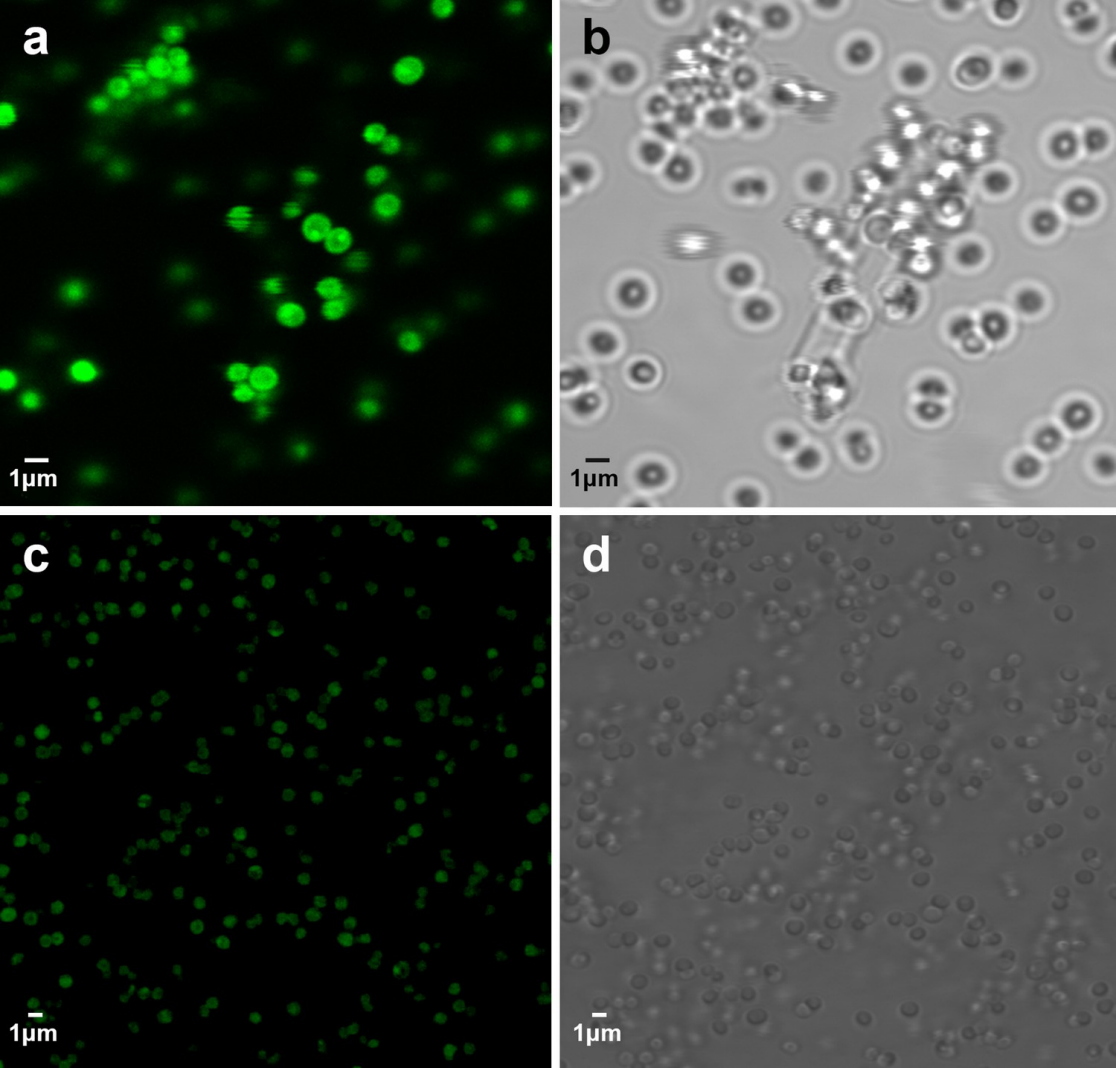
Fluorescence microscopy of bacteria with adherent cholesterol. At same light intensity confocal microscopy of *S*. *aureus* incubated with (a, b) and without (c, d) cholesterol crystals demonstrate binding of BODIPY stain to the bacteria exposed to cholesterol crystals but not in the bacteria that were not exposed to crystals.

*Confocal microscopy*. *S*. *aureus* bacteria were seen mainly in clusters. Only the bacteria exposed to CCs fluoresced green but not the control unexposed bacteria at the same light intensity (Fig 7).

## Discussion

To our knowledge, this is the first report to demonstrate a dynamic interaction between bacteria and CCs. This interaction has clinical implications with regards to bacterial endocarditis as well as the association of various systemic infections associated with heart attacks [5]. Adherence of bacteria to the cardiac valve surface is the initial event in the development of infective endocarditis. In experimental models of endocarditis studies have demonstrated that bacteria selectively adhere to traumatized valvular surfaces [16]. Our study investigated the effect of CCs on bacterial adherence and growth with a strong affiliation of both Gram positive and negative bacteria to CCs. Furthermore, we have recently demonstrated the presence of extensive CCs on heart valves from both human sclerotic valves and valves from an atherosclerotic rabbit model [9, 10]. Given the current study it becomes plausible that CCs can act as a nidus for bacteria to localize on the surfaces of abnormal valves. Staphylococcus and Gram-negative pathogens adhere to host cells and tissues via dedicated adhesion molecules [17]; however, the molecular nature of bacterial interactions with CCs has yet to be defined.

Cholesterol crystals have been found perforating the fibrous caps in patients who died with acute myocardial infarction but not in those with coronary atherosclerotic plaques and had died of other causes [8]. We have previously demonstrated that as cholesterol crystalizes it increases in volume causing the plaque core to expand making the sharp tipped CCs perforate the fibrous cap leading to plaque rupture [7, 8]. Other investigators have confirmed that when cholesterol crystallizes from a liquid to a solid state, it forms sharp tipped crystals that have the capacity to perforate fibrous tissue hence tearing the fibrous plaque cap [18]. Cholesterol crystals are known to be abundantly dispersed in atherosclerotic plaques. Also, bacteria have been found in atherosclerotic plaques however their role remains uncertain [19]. In this study we have demonstrated an affinity of *S*. *aureus* and *P*. *aeruginosa* to CCs *in vitro* and *ex vivo* with a significantly increased bacterial growth rate in the presence CCs. Persistent inflammation and mechanical injury associated with CC accretion within atherosclerotic plaques typically precedes plaque disruption (rupture and/or erosion) to cause thrombosis which is often the terminal events of atherosclerotic cardiovascular disease. In addition to the established role of CCs triggering a sterile inflammatory response, bacteria attached to the surface of the crystals could be an additional contributor to the inflammatory response further destabilizing atherosclerotic plaques.

In a previous study on aspirates from coronary arteries of patients during catheterization for myocardial infarction we had demonstrated that macrophages were attached to CCs and appeared to be binding and degrading them similar to what we found with bacteria in this study [11]. Also, similar findings were noted during CCs emboli in muscle [14]. Other reports have shown that *S*. *aureus* and *Mycobacterium tuberculosis* have the capacity to internalize and metabolize cholesterol thus utilizing free and esterified cholesterol as a potential source of nutrition and/or incorporation into the cell membrane as reported for mammalian cells [20, 21]. Specifically, in our study, both human carotid plaque and atherosclerotic rabbit arteries demonstrated a 3- and 4-fold increase of bacterial colony count respectively compared to normal arterial tissue. It is unlikely that the crystal adhesion to atherosclerotic plaque was related to the fibro-collagenous plaque cap since these plaques were totally eroded and had completely lost their fibrous caps as seen by SEM (Fig 6). This provides additional support for the potential role of CCs in not only injuring the valve tissue but also to act as an attractant for bacteria. Moreover, cholesterol seems to enhance bacterial resistance to antibiotics [22] while statins have been found to reduce inflammation and enhance wound healing during infections [23–25] suggesting that lowering LDLc as well as dissolving CCs with statins may contribute to reduction in available substrate for bacterial growth [26].

In our study we demonstrated that bacteria dissolved CCs and confocal microscopy demonstrated that bacteria exposed to CCs had stained green for the cholesterol dye, BODIPY, implying adherence of cholesterol to bacteria. This finding suggests that bacteria are able to degrade CCs [27, 28]. Thus, bacteria could be attracted to atherosclerotic plaques and cardiac valves with CCs contributing to plaque destabilization and seeding of sclerosed cardiac valves.

## Conclusions

This study demonstrates that bacteria associate with CCs *in vitro*, and *ex vivo* in rabbit and human arterial plaques rich in CCs. Recently we had demonstrated that CCs are found perforating the surface of cardiac valves in an atherosclerotic rabbit model and in sclerotic human valves. Since bacteria appear to have affinity to CCs, then it would be reasonable to assume that CCs can serve to adhere bacteria to valve tissue. Moreover, the presence of bacteria in atherosclerotic plaques has the potential of destabilizing the plaque elucidating the association of cardiovascular events following systemic infection. Further studies on the mechanism of interaction between bacteria and CCs are needed to explore these possibilities.

### Limitations

This study was conducted *in vitro* and *ex vivo* but not *in vivo*. Bacterial transcriptional responses were not evaluated to detect whether transcripts associated with cholesterol import or degradation were differentially regulated in response to CCs.

## Supporting information

S1 File (DOCX)

S2 File (DOCX)

S3 File (XLSX)
