# Supplementary material for: Interaction between bacteria and cholesterol crystals: Implications for endocarditis and atherosclerosis
Source: PLoS One. 2022 Feb 18;17(2):e0263847. doi: 10.1371/journal.pone.0263847 (PMC8856546; doi:10.1371/journal.pone.0263847)
Supplement: S1 File — (DOCX) [file pone.0263847.s001.docx]

**S1 Table. Summary statistics for adhesion studies *S. aureus***

| *Staphylococcus aureus* /10^3^ | | | | |
| --- | --- | --- | --- | --- |
|  | **Mean** | **Std Dev** | **Median** | **Interquartile**  **Range** |
| **Microspheres** | 55.53 | 43.29 | 51.50 | 48.70 |
| **CC** | 370.34 | 251.87 | 360.50 | 344.64 |
| **Glass** | 71.34 | 77.02 | 36.90 | 76.20 |
| **Glue** | 5.49 | 6.13 | 3.62 | 5.39 |

**S2 Table. Summary statistics for adhesion studies *P. aeruginosa***

| *Pseudomonas aeruginosa*/10^5^ | | | | |
| --- | --- | --- | --- | --- |
| **Group** | **Mean** | **Std Dev** | **Median** | **Interquartile**  **Range** |
| **Microspheres** | 23.02 | 43.83 | 7.86 | 11.76 |
| **CC** | 106.66 | 187.64 | 45.10 | 47.95 |
| **Glass** | 21.06 | 56.35 | 2.56 | 2.89 |
| **Glue** | 1.66 | 3.301 | 0.50 | 0.90 |

**S3 Table.** **Comparison of groups CC and microspheres***

|  | *Staphylococcus aureus* | | | *Pseudomonas aeruginosa* | | |
| --- | --- | --- | --- | --- | --- | --- |
| **Group** | **Estimate** | **95% Confidence Limits** | | **Estimate** | **95% Confidence Limits** | |
| **Microspheres** | 1.723 | 1.619 | 1.833 | 1.914 | 1.548 | 2.280 |
| **CC** | 2.352 | 2.245 | 2.460 | 3.620 | 3.254 | 3.986 |
| ***Difference*** | *0.627* | *0.475* | *0.778* | *1.707* | *1.189* | *2.224* |

* Transformed data. Row 1 and 2 entries are the time-averaged least-squares (LS-mean) estimates and associated 95% confidence limits. Row 3 is the difference in LS means and associated 95% confidence limits. The difference is significant (p<.0001) in both the *SA* and *PA* studies.

**S4 Table. Estimates and 95% confidence limits or mean bacterial colony growth***

|  | *Staphylococcus aureus* | | *Pseudomonas aeruginosa* | |
| --- | --- | --- | --- | --- |
| **Group** | **Estimate** | **95% Confidence**  **Limits** | **Estimate** | **95% Confidence**  **Limits** |
| **CC*** | 3.16 | 1.65, 4.66 | 2.37 | 2.05, 2.70 |
| **Control*** | 2.51 | 1.01, 4.01 | 1.86 | 1.54, 2.19 |
| **Ratio******, CC to Control** | 1.90 | 0.23, 15.91 | 1.67 | 1.05, 2.64 |

* Estimates and confidence limits are derived from transformed data. Row 1 and 2 entries are the time-averaged least-squares ( LS-mean) estimates and associated 95% confidence limits.

** Back-transformed to original scale.

**S5 Table. Summary statistics for bacterial colony count in rabbit model**

| **Rabbit Arteries** | **Incubation**  **Time, hours** | **Mean** | **Std Dev** | **Median** | **Interquartile**  **Range** |
| --- | --- | --- | --- | --- | --- |
| **Normal** | **1** | 500 | 418.33 | 400 | 200 |
|  | **3** | 2160 | 1409.96 | 1900 | 2200 |
| **Atherosclerotic** | **1** | 2100 | 815.48 | 1900 | 1400 |
|  | **3** | 12140 | 6116.62 | 13000 | 11200 |

**S6 Table. Summary statistics for bacterial colony count in human artery plaque samples***

| **Group** | **Mean** | **Std Dev** | **Median** | **Interquartile**  **Range** |
| --- | --- | --- | --- | --- |
| **Atherosclerotic** | 18.39 | 13.03 | 14.20 | 17.60 |
| **Control** | 5.91 | 6.44 | 4.70 | 7.00 |

* bacterial colony count/1000
