# Supplementary material for: Interaction between bacteria and cholesterol crystals: Implications for endocarditis and atherosclerosis
Source: PLoS One. 2022 Feb 18;17(2):e0263847. doi: 10.1371/journal.pone.0263847 (PMC8856546; doi:10.1371/journal.pone.0263847)
Supplement: S2 File — (DOCX) [file pone.0263847.s002.docx]

**Supporting Document - 3**

**BACTERIAL COLONY COUNT RAW DATA (BACTERIAL STUDIES.xlsx)**

**Growth Studies**

*P.aeruginosa* (PA) growth and *S.aureus* (SA) growth values Y1, Y2, Y3, Y4, Y5, Y6 are scaled by 10^3^. Data shown by GROUP and HOUR

**Adhesion Studies**

OUTCOME data are *SA* /10^3^ and *PA*/10^5^, shown by GROUP and HOUR for each REPLICATE.

**Rabbit artery study**

FEED: NF=normal feed, CF=cholesterol FEED

PERIOD: 1=Hour 1, 2=Hour 3

Y=bacterial colony count.

**Human artery plaque study**

RESPONSE data scaled by 10^3^, shown by GROUP and EXPT
